# The Effects of Microalgae as Live Food for *Brachionus plicatilis* (Rotifer) in Intensive Culture System

**DOI:** 10.21315/tlsr2018.29.1.9

**Published:** 2018-03-02

**Authors:** Abdull Razak Abd Rahman, Zaidi Che Cob, Zainoddin Jamari, Abdul Majid Mohamed, Tatsuki Toda, Othman Haji Ross

**Affiliations:** 1 Biology Unit, School of Applied Sciences, Universiti Teknologi MARA (UiTM), Perlis Branch, 02600 Arau, Perlis, Malaysia; 2 Marine-Science Programme, School of Environmental and Natural Resource Sciences, Universiti Kebangsaan Malaysia, 43600 UKM Bangi, Selangor, Malaysia; 3 Fisheries Research Institute (FRI), 11960 Batu Maung, Pulau Pinang, Malaysia; 4 Biology Unit, Centre for Foundation Science, Universiti Malaya, 50603 Kuala Lumpur, Malaysia; 5 Graduate School of Engineering, Soka University, Hachioji, Tokyo 192-8577, Japan; 6 Institute of Oceanography and Environment, and School of Marine and Environmental Sciences, Universiti Malaysia Terengganu, 21030 Kuala Terengganu, Terengganu, Malaysia

**Keywords:** *Brachionus placitilis*, Instantaneous Growth Rate, Intensive Culture, *Nannochloris* sp, *Tetraselmis* sp, *Isochrysis* sp, *Chlorella* sp., *Nannochloropsis* sp

## Abstract

*Brachionus plicatilis* is used to feed fish and crustacean larvae in the aquaculture industry. It is well established that the type of microalgae may influence rotifer production. This experiment was conducted to determine the effect of five different locally available microalgae species at Fisheries Research Institute (FRI), Kampung Pulau Sayak, Kedah, Malaysia on the instantaneous growth rate (μ) of rotifer. *Nannochloris* sp., *Tetraselmis* sp., *Isochrysis* sp., *Chlorella* sp., and *Nannochloropsis* sp. were used as feed at different algae densities (0.1, 0.3, 0.7 and 1.5 × 10^6^ cells/ml) and culture volumes (20, 70 and 210 ml). At algae densities ranging from 0.3 to 1.5 × 10^6^ cells/ml, an average μ value of more than 0.90 per day were recorded for all algae species. However, at density of 0.1 × 10^6^ cells/ml, only *Tetraselmis* sp. resulted in the significantly highest μ value compared with others (*p* < 0.05). In terms of volume, smaller culture volume of *Tetraselmis* sp. (20 ml) showed significantly higher μ compared with higher volume (70 and 210 ml cultures).

## Abstract (ru)

*Brachionus plicatilis* digunakan untuk memberi makan ikan dan larva krustacea dalam industri akuakultur. Telah termaktub bahawa jenis mikroalga boleh mempengaruhi pengeluaran rotifer. Eksperimen ini dijalankan untuk menentukan kesan spesis mikroalga yang terdapat di Institut Penyelidikan Perikanan (FRI), Kampung Pulau Sayak, Kedah, Malaysia pada kadar pertumbuhan segera (μ) rotifer. *Nannochloris* sp., *Tetraselmis* sp., *Isochrysis* sp., *Chlorella* sp. dan *Nannochloropsis* sp. digunakan sebagai makanan pada kepadatan alga yang berbeza (0.1, 0.3, 0.7 dan 1.5 × 106 sel/ml) dan jumlah kultur (20, 70 dan 210 ml). Pada kepadatan alga antara 0.3 hingga 1.5 × 106 sel/ml, nilai purata μ lebih daripada 0.90 sehari dicatatkan untuk semua spesies alga. Walau bagaimanapun, pada ketumpatan 0.1 × 106 sel/ml, hanya *Tetraselmis* sp. menghasilkan nilai μ yang paling tinggi berbanding dengan yang lain (*p* < 0.05). Dari segi isipadu, isipadu kultur *Tetraselmis* sp. yang lebih kecil (20 ml) menunjukkan μ yang lebih tinggi berbanding dengan isipadu kultur tertinggi (kultur 70 dan 210 ml).

## INTRODUCTION

The rotifer *Brachionus plicatilis* Muller, 1786 is one of the major food sources for fish and crustacean larvae in the aquaculture industry (Hoff & Snell 2001; Sorgeloos & Laven 1996; Lubzens *et al*. 1997; Lubzens & Zmora 2003). The rotifer has the ability to tolerate a wide range of salinity besides having the shape, size, colour, slow mobility and rapidly reproduce to reach high density in a short time, which contribute to the utilisation as initial food for the larvae. Furthermore the body composition of *B. plicatilis* can be manipulated to suit the nutritional requirements of the fish and crustacean larvae feeding on them (Lubzens *et al*. 1989).

The total length of *B*. *plicatilis* ranges between 100 to 400 μm and can be divided into three categories based, i.e. Type-L, 190–320 μm in length, Type-S, 140–220 μm and Type-SS, 100–160 μm (Hino & Hirano 1973). Type-SS was later known as *B. rotundiformis* (Segers 1995).

*B. plicatilis* reproduces asexually (parthenogenesis) under suitable condition by producing one or two large eggs of 80–100 × 110–130 μm every 4 to 6 hours and hatches to become an amictic female (Liao *et al*. 1993). In high density reproduction, in order to obtain mass production of rotifers, it is important to ensure that only asexual reproduction should prevail by avoiding factors that contribute to sexual reproductive phase (Liao *et al*. 1993; Theilacker & McMaster 1971). Factors such as high density of rotifers, food type, water temperature, salinity, light penetration, water quality and the genetic composition of rotifers can contribute to the types of reproduction of rotifers whether asexual or sexual reproduction (Hino & Hirano 1976, 1977; Lubzens *et al*. 1980).

One of the crucial problems in the production of fish fry in hatcheries is insufficient live food for the early stages of fish larvae and until now no alternative food can replace the use of rotifers as the initial food for larvae (Hagiwara *et al*. 2001; Yoshimatsu & Hossain 2014). Production of nutritious rotifers depends on the production of microalgae used to feed them. Apart from the nutritional quality, rotifer’s breeding rate is also strongly influenced by the species of microalgae given as food (Lubzens 1987; Lubzens *et al*. 2001).

Many species of microalgae have successfully been used as diet for rotifers such as *Tetraselmis*, *Nannochloropsis*, *Chaetoceros*, *Rhodomonas* and *Isochrysis* (Dhert *et al.* 2001; Hoff & Snell 2001; Treece & Davis 2000; Wikfors & Ohno 2001). Kostard *et al*. (1989a) reported higher growth rates when they were fed on *Isochrysis galbana* compared with *Tetraselmis*, *Nannochloris*, or mixture of *Isochrysis* and *Tetraselmis*, *Isochrysis* and *Nannochloris*, and *Tetraselmis* and *Nannochloris.* According to Sayegh *et al.* (2007), differences in strains of *Isochrysis galbana* used as feed resulted in some differences in the rotifer parameters such as growth rate, which was even greater than the differences of different diet species. Nevertheless, rotifers have different eating habits and varied structure and size of the corona, the mastax (specific pharynx) and also the mouth, which will determine the type of food that can be taken.

According to Yoshimatsu and Hossain (2014), various methods of microalgae cultures have been developed for successful production of rotifers, namely through batch-culture, semi-continuous culture and continuous culture system. To cater for the needs of live food for culturing fish larvae at high densities, rotifers can also be cultured under high density method. *Nannochloropsis* is the most common used as live food for rotifers because of its ease of culture and obtaining the yield expected with high density on time.

Mass scale production of rotifers comes with challenges of its own, such as drastic decrease in density due to the sudden collapse of rotifer cultures. In order to achieve high density and stability of rotifer cultures, microalgae play an important role as their food source. The Comparative study of the effects of microalgae *Nannochloris* sp., *Tetraselmis* sp., *Isochryris* sp., *Chlorella* sp. and *Nannochloropsis* sp*.* as live feed for rotifer on the growth rate of rotifer *B. plicatilis*is has yet to be determined in the confines of the Fisheries Research Institute (FRI). This study was aimed to examine the effectiveness of these species at different concentrations as live food and compared them with *Nannochloropsis* sp. because this algae is normally use in the hatchery. We assessed the effect of these differences by using a quantitative indicator (Instantanous growth rate of rotifer per day) and their relationship with the utilisation of different densities of food and volume of cultures.

## MATERIALS AND METHODS

A female amictic *Brachionus plicatilis* with loricae length of 110–230 μm was isolated from existing stock culture in FRI Pulau Sayak, which has been maintained for over a year. Rotifers culture was carried out using natural seawater at 30±2 psu, and fed *Nannochloropsis* sp. as the main food. The seawater used for culture medium was obtained from the coastal areas of Kg. Pulau Sayak, Kedah. The seawater was sterilised (autoclaved) before it was used to culture the rotifer, as well as the algae. The study was carried out entirely under laboratory conditions, at FRI Kg. Pulau Sayak, Kedah.

### Mass Culture of *Brachionus plicatilis* In Batch-culture System

One ml of stock culture, at density of 20 rotifers/ml was inoculated into a test-tube of 20 × 160 mm containing seawater at salinity 30±1 psu. The rotifers were fed *Nannochloropsis* sp. throughout the culture process at densities of 10–20 × 10^6^ cells/ml, which is the recommended density for intensive rotifer culture (Lee and Tamaru 1993). The rotifer culture was maintained at room temperature of 25±1°C and illumination of 150 lux. This starter cultures were then transferred to a 500 ml Erlenmeyer flasks to increase the volume, and again were fed, *Nannochloropsis* sp. at a concentration of 10–20 × 10^6^ cells per ml. The high density per ml of rotifer occurred in three days after inoculation. During this period there was no aeration given. Rotifers at exponential growth phase were then introduced into a 10 L aquarium filled with 5 L of seawater. Rotifers at this phase were cultured for three to four days before they were collected using a 30 μm mesh netting to be used in the experiments. The rotifers were always kept immersed in seawater during sieving.

### Live food preparation

Pure cultures of local strains of *Nannochloris* sp., *Tetraselmis* sp., *Isochrysis* sp., *Chlorella* sp. and *Nannochloropsis* sp. were obtained from FRI Pulau Sayak, and were cultured in batch-culture system. The microalgae range from 1–2 μm for *Nannochloris* sp., 9–10 μm×12–14 μm for *Tetraselmis* sp., 4–8 μm for *Isochrysis* sp., 2–10 μm for *Chlorella* sp., and 4–6 μm, for *Nannochloropsis* sp. The algal cultures were maintained in exponential growth phase using natural seawater at 30 psu and were enriched with Conway medium. In order to produce the inocula of pure strain algal for a larger volume, inoculum transfers were started from an axenic culture stock in a small volume culture. Contamination by bacteria normally cannot be avoided at a large volume culture, therefore the success of microalgae production depends on the inoculation procedure. The flasks cultures were kept on illuminated shelf of 3000 lux at room temperature of 24°C. Algal densities were estimated three times using a haemacytometer to obtain the average density to be used at the beginning for each feeding trials. All aseptic techniques were followed at every steps of sub-cultures transferred.

### Assessment of Live Feed for *Brachionus plicatilis*

The intensive cultures of *Brachionus plicatilis* were fed with *Nannochloris* sp., *Tetrasemis* sp., *Isochrysis* sp., *Chlorella* sp., and *Nannochloropsis* sp*.* separately and the instantaneous growth rate (μ) of the rotifers were determined. Prior to experiment, the rotifers were not fed for one day. Rotifers from the exponential growth phase were inoculated into a 50 ml Erlenmeyer flask filled with 20 ml of seawater at 30 psu. Assays were also performed at 70 and 210 ml volume cultures.

Each rotifer cultures were fed different microalgae species at densities of 0.1, 0.3, 0.7 and 1.5 × 10^6^ cells/ml. The algae densities used in this experiment were based on recommendations by Hoff and Snell (2001) for optimum feeding rate in rotifer. Test containers were exposed to fluorescent light intensity of 150 lux to maintain microalgae survival but prevent algal reproduction. No additional aeration was given. In this experiment all treatments were conducted in triplicate.

Summary of experimental design to assess live feed for *Brachionus plicatilis* as follows:

1. Microalgae – Batch culture system
2. Isolated Amictic Rotifer – Batch culture system
3. High Density of Rotifer (exponential growth phase)
4. Assessment of Live feed for *B. plicatilis*
  - Size of rotifer culture 20, 70 and 210 ml.
  - Each trail was fed different microalgae species at densities of 0.1, 0.3, 0.7 and 1.5 × 10^6^ cells/ml with triplicate
  - After 24 h, Sedgewick rafter slide used for counting rotifers and their eggs

### Rotifer Density Sampling

Triplicate of 1 ml sample from a homogenized rotifer culture were pipetted onto a sedgewick rafter slide using a micropipette. Counting of rotifers and their eggs were then conducted under a light microscope and their densities were determined. The number of rotifers and their eggs were summed up as the total number of rotifers. The instantanous growth rate (μ) was estimated after 24 h of treatments. The densities of rotifers and their eggs were determined as follows (Theilacker & McMaster 1971):

μ = (log_e_N_t_−log_e_N_o_)/t
N_o_ = The number of rotifers in the inoculation
N_t_ = The final number of rotifers after time t (days)

The growth rate of rotifer (μ) is calculated as the slope of log_e_ number of rotifers against time in days on the exponential growth phase.

### Statistical Test

SPSS package statististics 20.0 was used to examine the effects of the treatments on the parameters measured. In all analyses, the level of significance were considered at α = 0.05.

## RESULTS

The instantaneous growth rate of rotifers fed with *Nannochloris* sp. showed increased value with increasing food densities (Table 1). The highest values of instantanous growth rate were obtained at a density of 0.7 × 10^6^ cells per ml (1.53 per day) and was decreased at density of 1.5 × 10^6^ cells per ml, but showed no significant differences with density of 0.7 × 10^6^ cells per ml (Table 1). At density of 0.1 × 10^6^ cells per ml, the instantanous growth rate was only 0.10 per day.

The increment in instantaneous growth rate of rotifers fed on *Tetraselmis* sp. occurred by increasing food densities but had no significant effects (Table 1). The highest value of instantanous growth rate was occurred at density of 0.7 × 10^6^ cells per ml (1.72 per day) and the mean was decreased at density of 1.5 × 10^6^ cells per ml. At food density of 0.1 × 10^6^ cells per ml, the mean value already exceeded the μ values for other spp. (Table 1).

The instantaneous growth rate of rotifers per day fed on *Isochrysis* sp., increased with increasing food densities (Table 1). Even though the result showed a slightly lowered at density of 0.7 × 10^6^ cells per ml and the highest values of instantanous growth rate occurred at density of 1.5 × 10^6^ cells per ml (1.11 per day), however treatments at densities of 0.3 × 10^6^, 0.7 × 10^6^ and 1.5 × 10^6^ cells per ml indicated the mean values were not significant (Table 1). At density of 0.1 × 10^6^ cells per ml was 0.50 per day.

The instantaneous growth rate of rotifers per day fed on *Chlorella* sp. indicated increased value with increasing food densities (Table 1). At density of 0.1 × 10^6^ cells per ml, the mean value obtained only 0.24 per day. At density of 0.7 × 10^6^ cells per, showed significant increased in the mean value compared at density of 0.3 × 10^6^ cells per ml (Table 1). At density of 1.5 × 10^6^ per ml, showed the mean value was not significant compared to 0.7 × 10^6^ cells per ml (Table 1).

The instantaneous growth rate of rotifers per day fed on *Nannochloropsis* sp. also increased with increasing food densities (Table 1). At density of 0.3 × 10^6^ cells per ml, the mean value was achieved at 1.64 per day, but indicated that not significant to the densities of 0.7 × 10^6^ and 1.5 × 10^6^ cells per ml (Table 1). At density of 0.1 × 10^6^ cells per ml, the mean was only 0.10 per day.

At the level of food density 0.1 × 10^6^ cells per ml, among the five microalgae, *Tetraselmis* sp. resulted in significantly highest mean instantanous growth rates of rotifers (1.4 per day) (Table 2). The second highest was *Isochrysis* sp. (0.5 per day) and was significantly then those fed on *Nannochloris* sp., *Chlorella* sp*.*, and *Nannochloropsis* sp. (Table 2). Meanwhile, no differences were observed among *Nannochloris* sp., *Chlorella* sp*.*, and *Nannochloropsis* sp. for the values 0.1, 0.24 and 0.10 per day respectively. Meanwhile, at density of about 0.3 × 10^6^ cells per ml, all treatments recorded mean values higher per day. *Nannochloropsis* sp (1.64 per day) was higher than *Isochrysis* sp. and *Chlorella* sp., and *Tetraselmis* sp. (1.54 per day) was significantly different to *Chlorella* sp. (Table 2).

Diet at density of 0.1 × 10^6^ cells, *Tetraselmis* sp. capable to produce the highest instantanous growth rate per day of rotifers than those algal diet, treatments were repeated with the larger volume cultures (70 and 210 ml). The results were indicated that the greater volumes caused slower growth rate with significantly effects on volume culture 20 ml than volume cultures 70 ml and 210 ml (Table 3).

Experiments were conducted in larger volume cultures of rotifers at 70 ml, at the same level of food density. Statistical tests showed that the means of instantaneous growth rate of the rotifer fed on different diet species were not significantly differed (Table 4). Meanwhile, in culture volume of 210 ml, the instantaneous growth rates were 1.39 and 1.48 per day for *Tetraselmis* sp. and *Chlorella* sp. respectively showed significant difference to *Isochrysis* sp. (Table 4).

The effects of algal species were related to the densities of diet (two-way ANOVA; *p* < 0.01). The impacts of food densities (partial η^2^ = 0.48) are stronger than food species (partial η^2^ = 0.17; Table 5).

## DISCUSSION

Previous studies showed that the production of rotifers were affected by their food (Lubzens 1987; Lubzens *et al*. 2001). Sarma and Rao (1987) reported, the growth in many rotifers is a function of type of food and food levels provided. This was due to many factors that could influence the suitability of microalgae species as live food such as size, shape, mobility, digestibility and nutrient composition. However this study showed similar instantaneous growth rate when the algal densities were at the abundance level (0.3 × 10^6^ cells per ml). According to Yufera and Pascual (1980), the highest growth rate was recorded when using *Tetraselmis suecica*. Meanwhile Kostard *et al*. (1989a) reported highest growth rate when using *Isochrysis galbana* compared to others such as *Tetraselmis* sp., *Nannochloris atomus* or mixture of *Isochrysis* and *Tetraselmis*, *Isochrysis* and *Nannochloris*, and *Tetraselmis* and *Nannochloris*. *Isochrysis* sp. is a type of motile algae species, but with slow mobility compared to *Tetraselmis*. Although the use of *Tetraselmis* sp and *Isochrysis* sp. are ideal for rotifer, attaining high densitiy cultures are quite difficult compared with *Nannochloris*, *Nannochloropsis* and *Chlorella*.

Previous studies also reported strong correlation between rotifers growth rate and food abundance (Edmonson 1965; Hotos 2002; Snell *et al*. 1983). Lee and Tamaru (1993), suggest that food densities at 10 to 20 × 10^6^ cells per ml of *Nannochloropsis oculata* for intensive cultures of *Brachionus plicatilis*. In contrary, Hoff and Snell (2001), recommend a food density at only 0.1 × 10^6^ cells per ml for optimal nutrition values.

This study indicated at each of the four levels of food densities tested (i.e. 0.1 × 10^6^, 0.3 × 10^6^, 0.7 × 10^6^ and 1.5 × 10^6^ cells per ml) for all species resulted in instantaneous growth rates were higher except at the lowest food density tested at 0.1 × 10^6^ cells per ml for *Nannochloris* sp., *Isochrysis* sp., *Chlorella* sp. and *Nannochloropsis* sp.

At food density 0.1 × 10^6^ cells per ml, the mean instantaneous growth rates were depend on the food species. Only *Tetraselmis* sp. recorded significantly higher instantaneous growth rate. This indicated that at low food densities, the types of food become a limiting factor. Feature of *Tetraselmis* sp. that able to move faster than *Isochrysi*s sp. gave them advantage as preferred food for rotifers. This was followed by *Isochrysis* sp. with a mean value of the instantaneous growth rate at 0.50 per day and then by *Chlorella* sp., *Nannochloris* sp. and *Nannochloropsis* sp., which were non-motile species.

The mean instantaneous growth rates of rotifers increased significantly by increasing food densities from 0.1 × 10^6^ cells per ml to 0.3 × 10^6^ cells per ml, except *Tetraselmis* sp. Rotifers given food at density of 0.3 × 10^6^ cells per ml showed mean instantaneous growth rates were higher for all algae species. Therefore it was assumed that in intensive rotifers culture, higher density culture can be achieved at this level of algal density. The mean instantaneous growth rates were also higher at 0.7 × 10^6^ cells per ml and 1.5 × 10^6^ cells per ml. This indicated that rotifers were getting enough foods. However, *Tetraselmis* sp. still remains the preferred food. This study generally showed that foods given at densities of 0.1 × 10^6^ to 1.5 × 10^6^ cells per ml contributed to higher instantaneous growth rates for rotifers. Snell *et al*. (1983) reported, *B. plicatilis* recorded a peak in growth rate using *Chlorella* sp. at density of 0.5 mg per ml followed by a decrease in instantaneous growth rate at a high density of algae. Hotos (2002) stated the ingestion rate of *B. plicatilis* increased with increasing density of algae given and decreased at density 620,000 per ml. Meanwhile, Kostard *et al*. (1989b), observed ingestion rate by rotifers were linearly increased with an increase in food density until a maximum level and then remained constant.

The results indicated, when all species of food given at the abundance conditions produced higher impacts on the mean instantaneous growth rates of rotifers. But *Tetraselmis* showed better growth than *Chlorella*, *Nannochloropsis* showed better growth than *Isochrysis* and *Nannochloropsis* showed better growth than *Chlorella*. Rotifers reared at 70 ml and 210 ml culture volumes also did not revealed any significant differences, except for *Tetraselmis*-*Isochyris* and *Isochrysis*-C*hlorella* in 210 ml culture. There was no significant differences because the mean instantaneous growth rates using *Isochrysis* sp. was lower. The characteristic of motile preys can be ignored when at high density of food is given to rotifers, thus mobility factor of the preys only significant at low food density.

Our study suggest that culturing of rotifers in intensive culture systems can be conducted at food densities between 0.3 × 10^6^ cells per ml to 0.7 × 10^6^ cells per ml except for *Tetraselmis* sp., which can be supplied at lower density. All algal species tested at 1.5 × 10^6^ cells per ml showed declined pattern of rotifers growth rate, except for *Chlorella* sp..

Our study also revealed that culturing rotifers at high density were better when performed in small containers. Our result demonstrated that rotifers fed with *Tetraselmis* sp. at 0.1 × 10^6^ cells per ml, showed the highest mean instantaneous growth rate when culture in 20 ml volume culture (1.40 per day), and became lower in the 70 ml (0.80 per day) and 210 ml (0.60 per day) volume cultures. According to Rimatulhana *et al*. (2006), the growth rate for the *B. plicatilis* size SS, which was cultured in 750 ml at 30 psu using *Nannochloropsis* sp*.* at 3 × 10^6^ cells per ml was 0.787 per day. Yoshimatsu and Hossain (2014), reported in high density culture, the rotifers were exposed to a high pressure conditions such as from their own excreta, a situation that did not occur in their natural environment, and therefore will make the culture unstable.

Their study also showed the instantaneous growth rates of rotifers per day were higher when moving diets were used even at low food densities compared with the stationary food species that required higher densities for the same results. The use of *Tetraselmis* sp. and *Isochrysis* sp. at lower densities than the three other species produced a higher instantaneous growth rate of rotifers. Factor of mobility for *Tetraselmis* sp. and *Isochrysis* sp. were met with their predators become more frequent.

Statistical test revealed that the effects of food densities on the mean instantaneous growth rate values were varied between species (*p* < 0.01) and furthermore the food densities dominated over food species on rotifers production. According to Theilacker and McMaster (1971), high food densities are the most important parameters needed to ensure high production of rotifer**.**

## CONCLUSION

Generally, the instantaneous growth rate of rotifers per day increased with increasing food densities. All food species assayed at density of 0.3 × 10^6^ cells per ml, showed all the mean values of instantaneous growth rates were higher when the algal densities at the abundance level (0.3 × 10^6^ cells per ml). The type of microalgae also can become a limiting factor which at food density of 0.1 × 10^6^ cells per ml (recommended by Hoff and Snell 2001), showed only food species have the capability to move (motile food species) such as *Tetraselmis* sp. and *Isochrysis* sp. produced the high instantaneous growth rates. This study also shown that the smaller volume culture size has a better result for mass production of rotifers.

## Figures and Tables

**Table 1:** Mean value with standard error of mean of instantaneous growth rate of rotifer (μ/day) fed on different densities of microalgae in 20 ml culture volume. The symbol (*) and dissimilar alphabet (superscript) in row denote differences at α = 0.05.

Diet	Density (cell/ml)				*p* value

	0.1 × 10^6^	0.3 × 10^6^	0.7 × 10^6^	1.5 × 10^6^	
Nris	0.10^a^±0.00	1.16^b^±0.21	1.53^b^±0.07	1.28^b^±0.25	0.01^*^
Tetra	1.40^a^±0.08	1.54^a^±0.21	1.72^a^±0.09	1.62^a^± 0.21	0.60
Iso	0.50^a^±0.10	1.04^b^±0.13	0.98^b^±0.09	1.11^b^±0.15	0.03^*^
Chl	0.24^a^±0.14	0.89^b^±0.09	1.41^c^±0.00	1.67^c^±0.13	0.02^*^
Nanno	0.10^a^±0.00	1.64^b^±0.14	1.27^b^±0.37	1.33^b^±0.22	0.07^*^

Nris *= Nannnochloris s*p.; Tetra *=Tetraselmis* sp.; Iso *= Isochrysis* sp.; Chl *= Chlorella* sp.; Nanno *= Nannochloropsis* sp.

**Table 2:** Mean value with standard error of mean of instantaneous growth rate of rotifer (μ/day) fed on different diets at two different densities in 20 ml culture volume. The symbol (*) and dissimilar alphabet (superscript) in row denote differences at α = 0.05.

Density (cell/ml) × 10^6^	Diet					*p* value

	Nris	Tetra	Iso	Chl	Nanno	
0.1	0.10^a^±0.00	1.40^b^±0.08	0.50^c^±0.10	0.24^a^±0.14	0.10^a^±0.00	0.02^*^
0.3	1.17^abc^±0.22	1.54^bc^±0.21	1.04^ac^±0.13	0.89^a^±0.50	1.64^b^±0.14	0.04^*^

**Table 3:** Fisher LSD test results of instantaneous growth rate of rotifers per day at *Tetraselmis* sp. density of 0.1 × 10^6^ cells/ml. Asterisk denote differences at α= 0.05.

Culture volume (mL)	*p*
20–70	0.001**
20–210	0.001**
70–210	0.080^ns^

ns = Not significant;

** *p* < 0.01

**Table 4:** Mean value with standard error of mean of instantaneous growth rate of rotifer (μ/day) fed at 0.3 × 10^6^ cells/ml on different diets in 70 and 210 ml culture volumes. The dissimilar alphabet (superscript) in row denote differences at α = 0.05.

Culture volume (ml)	Diet					*p* value

	Nris	Tetra	Iso	Chl	Nanno	
70	0.80^a^±0.00	0.80^a^±0.00	0.90^a^±0.10	1.27^a^±0.33	0.80^a^±0.00	0.17
210	1.09^ab^±0.05	1.39^b^±0.05	0.60^a^±0.09	1.48^b^±0.41	1.02^ab^±0.29	0.12

**Table 5:** Two-way ANOVA of diet and density effects on instantaneous growth rate of rotifers per day

Source	*Df*	*SS*	*MS*	*F*	Partial η^2^
Species (S)	4	3.156	0.789	10.048**	0.17
Density (D)	3	8.853	2.951	37.578**	0.48
S × D	12	3.304	0.275	3.506**	0.18
Error	40				
